# A case of perirenal non‐specific lymphadenitis mimicking a solitary renal mass

**DOI:** 10.1002/iju5.12371

**Published:** 2021-09-09

**Authors:** Shun Umeda, Koji Hatano, Taigo Kato, Atsunari Kawashima, Toyofumi Abe, Shinichiro Fukuhara, Motohide Uemura, Hiroshi Kiuchi, Ryoichi Imamura, Norio Nonomura

**Affiliations:** ^1^ Department of Urology Osaka University Graduate School of Medicine Suita Japan

**Keywords:** biopsy, kidney, laparoscopic surgery, lymphadenitis, lymphadenopathy

## Abstract

**Introduction:**

Since the diagnosis of small renal masses is often a challenge despite improvements in imaging modalities, renal tumor biopsy provides useful information regarding treatment decisions. However, there is no established treatment strategy when renal biopsy shows lymphoid tissue.

**Case presentation:**

A 63‐year‐old woman was referred to our department for the investigation of a small renal mass. Contrast‐enhanced computed tomography showed a weakly enhancing mass 39 × 17 mm in diameter in the left kidney. A renal tumor biopsy was performed, and histopathological examination showed lymphoid tissue, but the diagnosis was not confirmed. The tumor was bluntly dissected from the renal capsule via robotic‐assisted laparoscopic surgery without renal artery clamping. The pathological diagnosis was non‐specific lymphadenitis.

**Conclusion:**

We report a rare case of perirenal non‐specific lymphadenitis mimicking a solitary renal mass. Non‐specific lymphadenitis is a possible differential diagnosis of renal masses.

**Abbreviations & Acronyms**
CMV: cytomegalovirus
CT: computed tomography
EBER‐ISH: Epstein‐Barr virus‐encoded RNA in situ hybridization
HHV: human herpesvirus
HSV: herpes simplex virus
RCC: renal cell carcinoma

**Keynote message**

Confirmation of the pathological diagnosis is challenging when renal mass biopsy shows lymphoid tissue. Surgical resection could be a therapeutic option for a solitary perirenal lymphoid mass and lead to a definitive pathological diagnosis.

## Introduction

Small renal masses represent a heterogeneous group of tumors, including RCC, benign renal lesions, and rarely, lymphoid tissue. Renal mass biopsy provides useful information to guide treatment selection in patients with small renal masses.^1^ To date, the clinical management of a solitary lymphoid renal mass has not yet been established.

## Case presentation

**Fig. 1:**
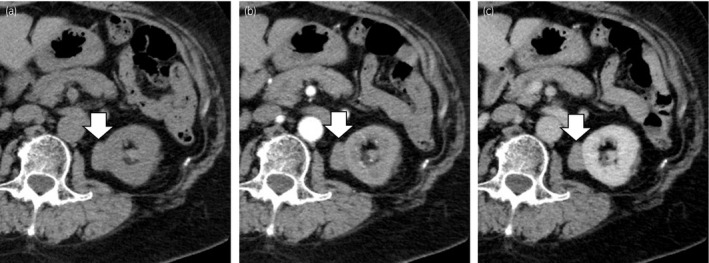
Enhanced abdominal CT shows a slowly and weakly enhancing mass 39 × 17 mm in diameter in the left kidney; (a) plain, (b) early phase, (c) late phase. Arrows: tumor.

A 63‐year‐old woman presented to her family doctor with a complaint of asymptomatic gross hematuria. Cystoscopy showed hematuria originating from the left ureteral orifice, and CT showed a left renal mass. The patient was referred to our hospital for further investigation. At the time of the initial examination, the patient had no clinical symptoms. All laboratory analyses were normal, and urine occult blood test results were negative. Contrast‐enhanced CT revealed a slowly and weakly enhancing mass 39 × 17 mm in diameter in the left kidney (Fig. 1). Based on the imaging findings, we suspected papillary RCC or metanephric adenoma.

**Fig. 2:**
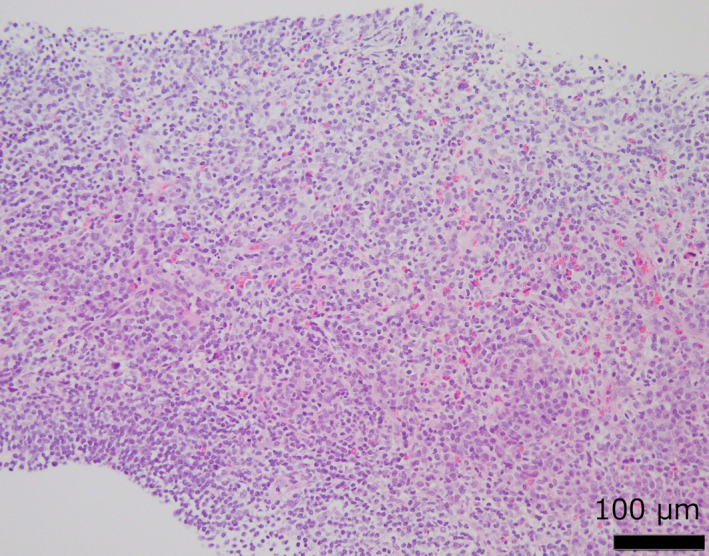
Histopathological findings of the renal tumor biopsy. Hematoxylin and eosin staining shows dense growth of small‐to‐medium‐sized lymphocytes. Scale bar: 100 μm.

We performed percutaneous renal tumor biopsy because the imaging findings were not typical for clear cell RCC. On hematoxylin‐eosin staining, small‐to‐medium‐sized lymphocytes were densely congregated, but cellular atypia was not observed (Fig. 2). Immunohistochemical staining was partially positive for B‐cell lymphoma 6 (BCL‐6), octamer‐binding transcription factor 2 (OCT‐2), and programmed cell death 1 and negative for terminal deoxynucleotidyl transferase, CD10, CD21, and CD30. Clonality analysis revealed no monoclonal rearrangements. EBER‐ISH was negative. Since a lymphoid malignancy could not be ruled out and the tumor was thought to be of renal origin on imaging, we decided to perform a robot‐assisted partial nephrectomy. soluble interleukin‐2 receptor was not measured.

**Fig. 3:**
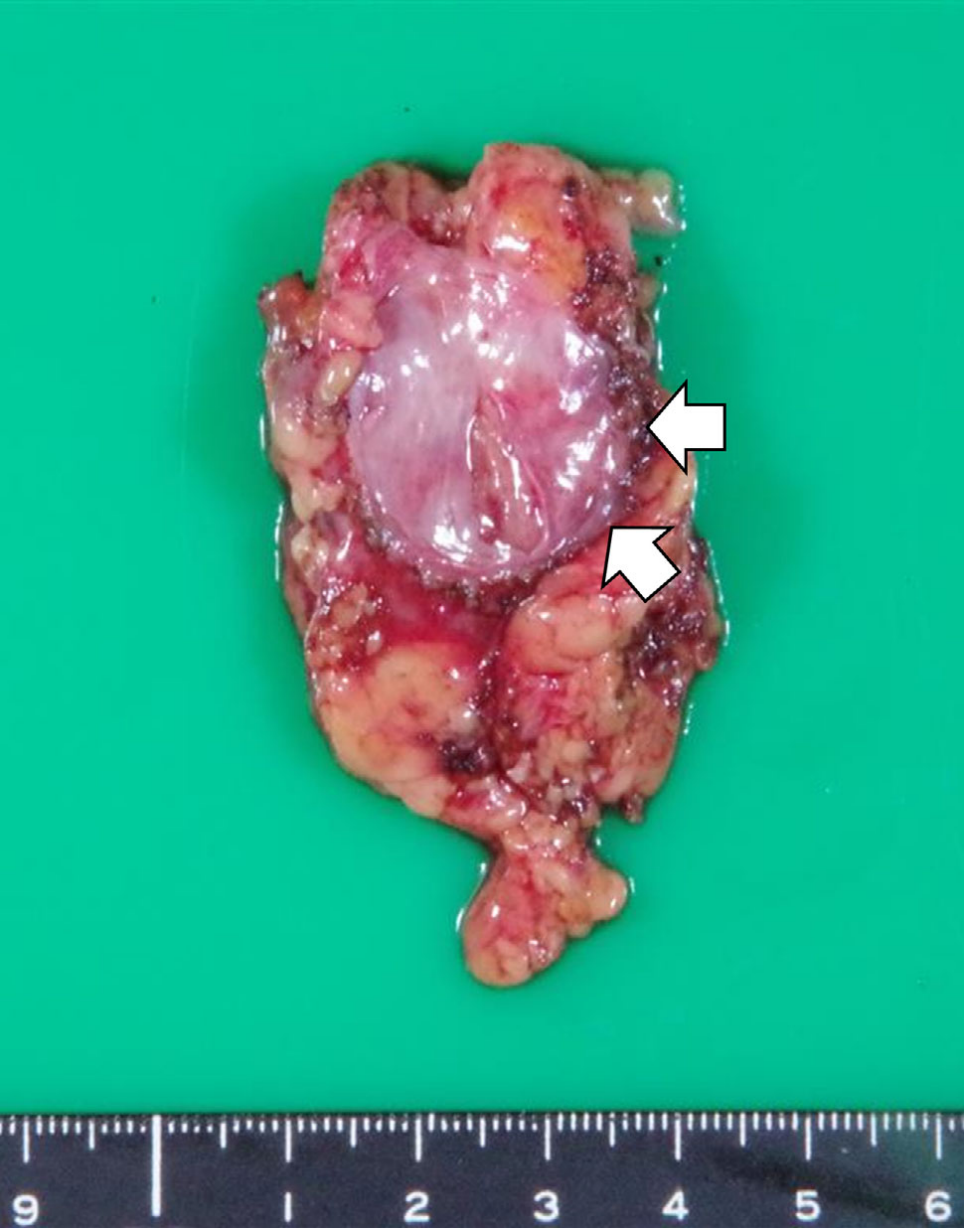
Macroscopic finding shows a well‐circumscribed and uniform yellowish‐white mass 3 cm in diameter. Arrows: tumor.

**Fig. 4:**
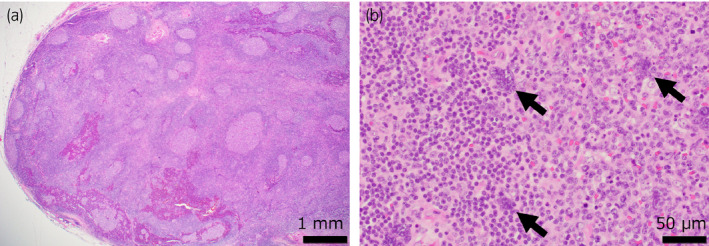
Histopathological findings of the specimen. (a) In low power field, secondary lymphoid follicles are conspicuous, and the basic structure of the lymphoid tissue is generally preserved. Scale bar: 1 mm. (b) In high power field, large, multinucleated cells are observed (arrows). Scale bar: 50 μm.

The surgery was performed transperitoneally, with an operative time of 223 min, a console time of 96 min, and a blood loss of 20 mL. The mass was bluntly dissected from the renal capsule without renal artery clamping. There were no perioperative complications. Gross pathology revealed a well‐circumscribed and uniform yellowish‐white mass 3 cm in diameter (Fig. 3). Microscopically, secondary lymphoid follicles were conspicuous, and the basic structure of the lymphoid tissue was generally preserved (Fig. 4a). There were no caseating granulomas. A characteristic finding was the presence of large multinucleated cells, suggestive of viral infection (Fig. 4b). However, virus infection markers, including HHV8, HSV, CMV, and EBER‐ISH, were negative. Immunohistochemical staining was partially positive for BCL‐6 and negative for OCT‐2, PAX5, STAT6, multiple myeloma oncogene 1, B cell OCT binding protein 1, CD3, CD15, CD20, CD30, CD56, CD79a, TCRβF1, TCRδ, programmed‐death ligand 1 (PD‐L1), and leukocyte common antigen. Clonality analysis did not reveal any monoclonal rearrangements. Based on these results, we diagnosed the patient with non‐specific lymphadenitis. Seven months after surgery, no recurrence has been observed.

## Discussion

Lymphoid masses such as Castleman's disease, inflammatory pseudotumor, and malignant lymphoma are known to occur in the kidney.^2^, ^3^, ^4^ Kikuchi‐Fujimoto disease, which causes subacute necrotizing lymphadenopathy around the kidney, is observed in young adults of Asian descent.^5^ Non‐specific lymphadenitis is common in the superficial lymph nodes and accounts for 22% of cervical lesions.^6^ To the best of our knowledge, there are only two reports of perirenal non‐specific lymphadenitis mimicking a renal mass, including the present case. Kubota *et al*. reported a case of non‐specific lymphadenitis presenting as a 30‐mm renal mass resected via laparoscopic partial nephrectomy.^7^ Perirenal non‐specific lymphadenitis is characterized by benign tumors that can be easily dissected, pathologically preserved basic structure of lymphoid tissue, the presence of large multinucleated cells, and no evidence of specific viral infection.

The diagnosis of small renal masses is often challenging despite improvements in imaging modalities such as CT and magnetic resonance imaging. Recent studies reported that 6.9–31.0% of patients who underwent laparoscopic partial nephrectomy had benign tumors.^8^, ^9^, ^10^, ^11^, ^12^ Snyder *et al*. reported that 16.4% of 815 renal tumors resected by nephrectomy were benign, with 10.7% being oncocytoma, 2.0% angiomyolipoma, 1.2% simple cysts, 1% metanephric adenoma, and 0.6% cystic nephroma.^13^ Nishikawa *et al*. reported that 30.1% of cases were benign in the group with atypical imaging pattern for clear cell RCC on enhanced CT, although 6.9% of cases were benign in the group with typical imaging patterns for clear cell RCC.^12^ Therefore, the accuracy of imaging diagnosis is limited for small renal tumors, especially when contrast‐enhanced CT does not show typical clear cell RCC findings. In such cases, renal tumor biopsy provides useful information for treatment decisions.

Surveillance can be an option when renal biopsy shows a benign renal tumor in order to avoid unnecessary surgical procedures. However, when biopsy results show lymphoid tissue, it is difficult to distinguish malignant diseases, such as lymphoma, from benign lymphadenopathy.^14^ Groneck *et al*. reported that the positive predictive value of core needle biopsy of lymph nodes is 89% for non‐Hodgkin's lymphoma and 44% for Hodgkin's lymphoma for the diagnosis of lymphadenopathy in the cervical, axillary, inguinal, or other subcutaneous regions.^15^ Consequently, 54 of 121 patients underwent secondary biopsy for confirmation of the diagnosis, and 26 patients underwent surgical resection for confirmation of the pathological diagnosis.^15^ Therefore, it is difficult to confirm the pathological diagnosis of lymphoid tumors based on the initial biopsy, necessitating secondary biopsy, surgical resection, or careful follow‐up.^15^ In addition, it is possible that lymphadenitis coexists with malignant lymphoma in the same lymph node.^16^ Focal therapy for perirenal lymphatic tumors has not been currently established.

In our case, the lymphadenopathy was solitary and located adjacent to the renal capsule, so it could be resected safely with minimal invasiveness without renal artery clamping. Therefore, the preoperative tumor biopsy would be useful for selecting between lymphadenectomy and partial nephrectomy.

## Conclusion

We report a case of a solitary perirenal mass with a histopathological diagnosis of non‐specific lymphadenitis after laparoscopic resection. Non‐specific lymphadenitis is a possible differential diagnosis of lymphoid renal masses.

## Conflict of interest

The authors declare no conflict of interest.

## Approval of the research protocol by an Institutional Reviewer Board

Not applicable.

## Informed consent

All human subjects provided written informed consent with guarantees of confidentiality.

## Registry and the Registration No. of the study/trial

Not applicable.
